# Variation of Activation Volume as an Indicator of the Difference in Clusterization Phenomenon Induced by H-Bonding and F−Π Stacking Interactions in Enantiomers and a Racemate of Flurbiprofen

**DOI:** 10.1021/acs.jpcb.4c00582

**Published:** 2024-04-12

**Authors:** Paulina Jesionek, Barbara Hachuła, Karolina Jurkiewicz, Patryk Włodarczyk, Marek Hreczka, Kamil Kamiński, Ewa Kamińska

**Affiliations:** † Institute of Chemistry, Faculty of Science and Technology, University of Silesia in Katowice, Szkolna 9, 40-007 Katowice, Poland; ‡ Department of Pharmacognosy and Phytochemistry, Faculty of Pharmaceutical Sciences in Sosnowiec, Medical University of Silesia in Katowice, Jagiellonska 4, 41-200 Sosnowiec, Poland; § Institute of Physics, Faculty of Science and Technology, University of Silesia in Katowice, 75 Pulku Piechoty 1, 41-500 Chorzow, Poland; ∥ Łukasiewicz Research Network - Institute of Non-Ferrous Metals, Sowinskiego 5 St., 44-100, Gliwice, Poland; ⊥ Department of Mechatronics, Silesian University of Technology, Akademicka 10A St., 44-100 Gliwice, Poland

## Abstract

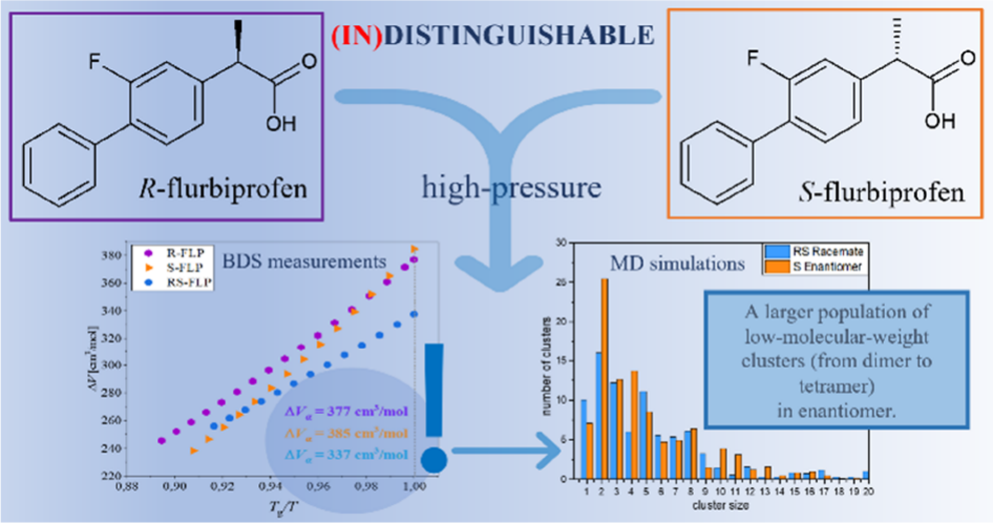

In this paper, X-ray diffraction (XRD), differential scanning calorimetry (DSC), broadband dielectric (BDS), and Fourier transform infrared (FTIR) spectroscopy supported by molecular dynamics (MD) simulations and quantum chemical computations were applied to investigate the structural and thermal properties, molecular dynamics, and H-bonding pattern of *R*-, *S*-, and *RS*-flurbiprofen (FLP). Experimental data indicated various spatial molecular arrangements in crystalline forms of examined systems, which seemed to disappear in the liquid state. Surprisingly, deeper analysis of high-pressure dielectric data revealed unexpected variation in the activation volume of pure enantiomers and a racemate. MD simulations showed that it is an effect of the clusterization phenomenon and a higher population of small associates in the former samples. Moreover, theoretical consideration exposed the particular role of unspecific F−Π interactions as a driving force underlying local molecular arrangements of molecules in the liquid and the crystal lattice of *R*-, *S*-, and *RS*-FLP.

## 1 Introduction

In recent years, enantiomers, also called optical isomers or chiral molecules, have been the subject of intensive research in biology, chemistry, physics, and pharmaceutical sciences.^1−10^ These systems are indistinguishable from each other regarding most physicochemical properties, such as chemical formula, melting points, or glass-transition temperature (*T*_g_), except for the direction of their refraction of plane-polarized light^6^ or circular dichroism.^11^ Nevertheless, many papers have demonstrated that despite such strong similarity of enantiomers, they can vary significantly in their biological, toxicological, as well as pharmacodynamic, and pharmacokinetic properties.^6,8,12−23^

One of the groups of active pharmaceutical ingredients (APIs), where chirality seems to be an important aspect that is systematically and thoroughly studied from physical, chemical, and pharmaceutical perspectives, are 2-arylpropionic acids, the so-called profens.^24−36^ The presence of a carboxylic moiety capable of forming hydrogen (H)-bonds in the close vicinity of the chiral carbon atom makes the situation very interesting since chirality can influence spatial molecular organization, self-assembly phenomenon, etc. This supposition was positively verified by studying, e.g., the H-bond pattern in the crystalline pure *R*- and *S*-enantiomers of flurbiprofen (FLP) and the racemic (*RS*) mixture. Namely, in the former systems, the chain-like organization of the molecules was found, while in the latter, dimeric structures were preferred.^27^ Interestingly, the case seems to be much different in the supercooled liquid state. Molecular dynamic (MD) simulations carried out by Ottou Abe et al.^34^ on ibuprofen (IBP) showed that the population of H-bonded (HB) aggregates, as well as the proportion of cyclic vs linear dimers, are comparable for pure *S*-enantiomer and the *RS*-racemate. Moreover, it is worth mentioning the investigations by Adrjanowicz et al., which revealed that structural (α)-relaxation times, as well as diffusion of pure *S*-enantiomer and *RS*-racemate of ketoprofen (KTP), are very similar around the *T*_g_.^36^ Herein, one can also briefly refer to the studies on the chiral compound from a different group, i.e., *N*-acetyl-α-methylbenzylamine (strictly R(+) and S(−) enantiomers, conglomerates at various enantiomeric excesses (ee) and the racemic mixture (ee = 0%)). It was presented that also the time scales of α-relaxation (and other processes detected in BDS spectra, i.e., Debye, β, and γ), as well as their temperature dependences, are insensitive to chirality. However, factors such as glass-forming ability and the recrystallization of heterochiral arrangements turned out to be chirality-dependent.^37^

The behavior of the enantiomers and racemates was also studied at high pressure. It was shown that it is possible to separate pure enantiomers from the racemic mixture after crystallization of the latter systems in the liquid phase at high compression.^38−40^ However, such an approach can be effective only when both chiral molecules are denser than the racemate.^41^ The other interesting high-pressure studies on racemates (profens) were performed by Adrjanowicz et al.^26,36^ They indicated that there is only a small difference in the structural dynamics of the *S*-enantiomer of KTP and the racemic (*RS*) mixture under various temperature and pressure conditions. As a consequence, the pressure coefficient of the glass-transition temperature (d*T*_g_/d*p* = 0.193 and 0.200 K/MPa, respectively), as well as the activation volume of both systems, were almost the same.^36^ Furthermore, isochronal crystallization (τ_α_ = const) at high pressure revealed that the *S*-enantiomer of KTP forms crystals much faster with respect to the racemic mixture.^36^ A different scenario was noted in the case of IBP. Koperwas et al.^42^ assigned the observed discrepancy (i.e., a greater slowing down of crystallization in the case of pure *S*-isomer compared to the racemate) to a distinct impact of compression on the solid–liquid interfacial free energy of both systems.

This review presents the results of structural, spectroscopic, thermal, and dielectric studies carried out at ambient and elevated pressure on *R-* and *S-*enantiomers of FLP and their racemic mixture. The purpose of these studies, supported by molecular dynamics (MD) simulations and density functional theory (DFT) computations, was to answer the question of whether there are differences in molecular organization via H-bonds in pure enantiomers and the racemate in the crystalline and supercooled liquid states. If so, what do they result for? Moreover, the intention was to verify if the atomic structure, thermal properties, and ambient and high-pressure molecular dynamics of neat *R*- and *S*-FLP are similar to those of *RS*-FLP.

## 2 Experimental Section

### 2.1 Materials

*R*- and *S*-FLP, as well as their racemic mixture (*RS*-FLP) with a purity higher than 98%, were supplied by MedChemExpress and Acros Organics Chemicals, respectively, and used without further purification.

### 2.2 Methods

#### 2.2.1 X-ray Diffraction (XRD)

XRD patterns of crystalline and molten FLP samples were collected on a Rigaku Denki D/Max Rapid II diffractometer equipped with a 2D image plate detector and a rotating Ag anode. Incident radiation was monochromatized, and the wavelength of the beam was λ_*K*_α1,α2__ = 0.5608 Å. The temperature was controlled by an Oxford Cryostrem system. Samples were measured in borosilicate glass capillaries, and the background from the empty capillary was subtracted.

#### 2.2.2 Fourier Transform Infrared (FTIR) Spectroscopy

FTIR spectra of crystalline samples of racemic and chiral forms of FLP were recorded with a Nicolet iS50 spectrometer (Thermo Scientific) equipped with an attenuated total reflectance (ATR) accessory in the 4000–400 cm^–1^ region at 293 K. They were obtained with 16 scans and a resolution of 4 cm^–1^. The spectra were also collected on a Nicolet iS50 spectrometer coupled with a Pike GladiATR module at 403 K. The measurements were taken with 16 scans and a resolution of 2 cm^–1^ in the wavenumber range of 4000–400 cm^–1^.

#### 2.2.3 Differential Scanning Calorimetry (DSC)

Calorimetric experiments were performed using a Mettler-Toledo DSC apparatus equipped with a liquid nitrogen cooling accessory and an HSS8 ceramic sensor with 120 thermocouples. The instrument was calibrated for temperature and enthalpy using indium and zinc standards. The samples were placed in sealed aluminum crucibles (40 μL), then heated above their melting temperatures, quenched, and scanned at the rate of 10 K/min well above the respective melting points.

#### 2.2.4 Broadband Dielectric Spectroscopy (BDS)

Isobaric measurements of the complex dielectric permittivity ε*(ω) = ε′(ω) – iε″(ω) (frequency range from 10^–2^ to 10^6^ Hz) were performed using a Novocontrol α dielectric spectrometer equipped with α impedance analyzer with an active sample cell. The samples were placed in a parallel-plate cell made of stainless steel (diameter: 15 mm; gap: 0.1 mm with Teflon spacer), mounted inside a cryostat, and kept under dry nitrogen gas flow during measurements. The temperature control was provided by a Quatro cryosystem with temperature stability better than 0.1 K. Measurements were carried out after fast cooling the liquid to the glassy state and were done in the temperature range from 298 to 203 K.

For dielectric studies at elevated pressure, a high-*p* chamber with a special homemade flat parallel capacitor was used. Thin Teflon spacers were employed to maintain a fixed distance between the plates. Each sample capacitor was sealed and mounted inside a Teflon capsule to separate it from the silicon liquid (a pressure-transmitting medium). Pressure was measured using a Nova Swiss tensometric meter with a resolution of 1 MPa. In turn, the temperature was adjusted with a precision of 0.1 K by means of a refrigerated and heated Huber circulator. Measurements of complex dielectric permittivity were performed in the same frequency range as in the case of measurements carried out at *p* = 0.1 MPa. It should be noted that each dielectric experiment (including sample preparation) was repeated three times. For more detailed information about the BDS technique (both ambient and high-pressure studies), see refs (43−45)

#### 2.2.5 Molecular Dynamics (MD) Simulations

Molecular dynamics simulations were performed in the Gromacs 2023 package^46−50^ using a Gromos 54a7^51^ force field. The topology for both FLP molecules was created by an online automated topology builder (ATB).^52−54^ Starting geometry was generated by Packmol.^55^ In the case of the *S*-enantiomer, it was 500 *S*-FLP molecules, while in the case of the racemate, it was 250 of *S*- and 250 of *R*-molecules. After the initial geometry optimization, two subsequent equilibrating simulations were performed. They were carried out in the NPT (constant temperature, constant pressure) ensemble, where the v-rescale and c-rescale were used as thermostat and barostat, respectively. The pressure was set in both simulations to 1 bar, while the temperature was set to 400 K in the first simulation and 293 K in the second simulation. The final production run was done as a 10 ns simulation in the NPT ensemble with *p* = 1 bar and *T* = 293 K using the same type of thermal and pressure control. The analysis of the production runs was performed using the Travis package,^56−58^ which enabled the provision of all needed RDFs, ADFs, and HB topologies. The histogram of the cluster sizes was obtained by the use of the Gromacs “clustsize” routine.

#### 2.2.6 Density Functional Theory (DFT) Calculations

DFT computations were carried out in the ORCA 5.0.4 package.^59−63^ The starting geometry for calculating the *R*-*S* Π-stacking interaction was obtained from a cif file for the racemate. Then, the chirality of one molecule was changed by switching the H and OH group positions to form *S–S* Π-stacking interacting molecules. The geometry optimization was performed at the B97M-D4/6-31G(2d,2p) level of theory. The starting geometry of the dimers with linear HBs was created in a few steps. In the first step, the molecular dynamics algorithm in orca was employed to obtain 100 random dimeric structures by performing simulation at *T* = 293 K and saving xyz file after each 0.1 ps of simulation (where total time was 10 ps). All saved geometries were optimized in the xtb semiempirical method, and then, the 20 most energetically stable forms were optimized in the DFT method on the B97M-D4/6-31G(2d,2p) level of theory. The interaction energies were calculated as a difference between the energy of the optimized dimer and the sum of the energy of isolated molecules, according to the equation
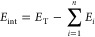
1where *E*_int_ is the interaction energy, *E*_T_ is the total energy of the entire complex, and *E*_i_ is the energy of every isolated molecule forming the complex.

All of the energies were calculated at the B97/6-311G(2df,2pd) level of theory using additional D4 corrections.

## 3 Results and Discussion

**Figure 1:**
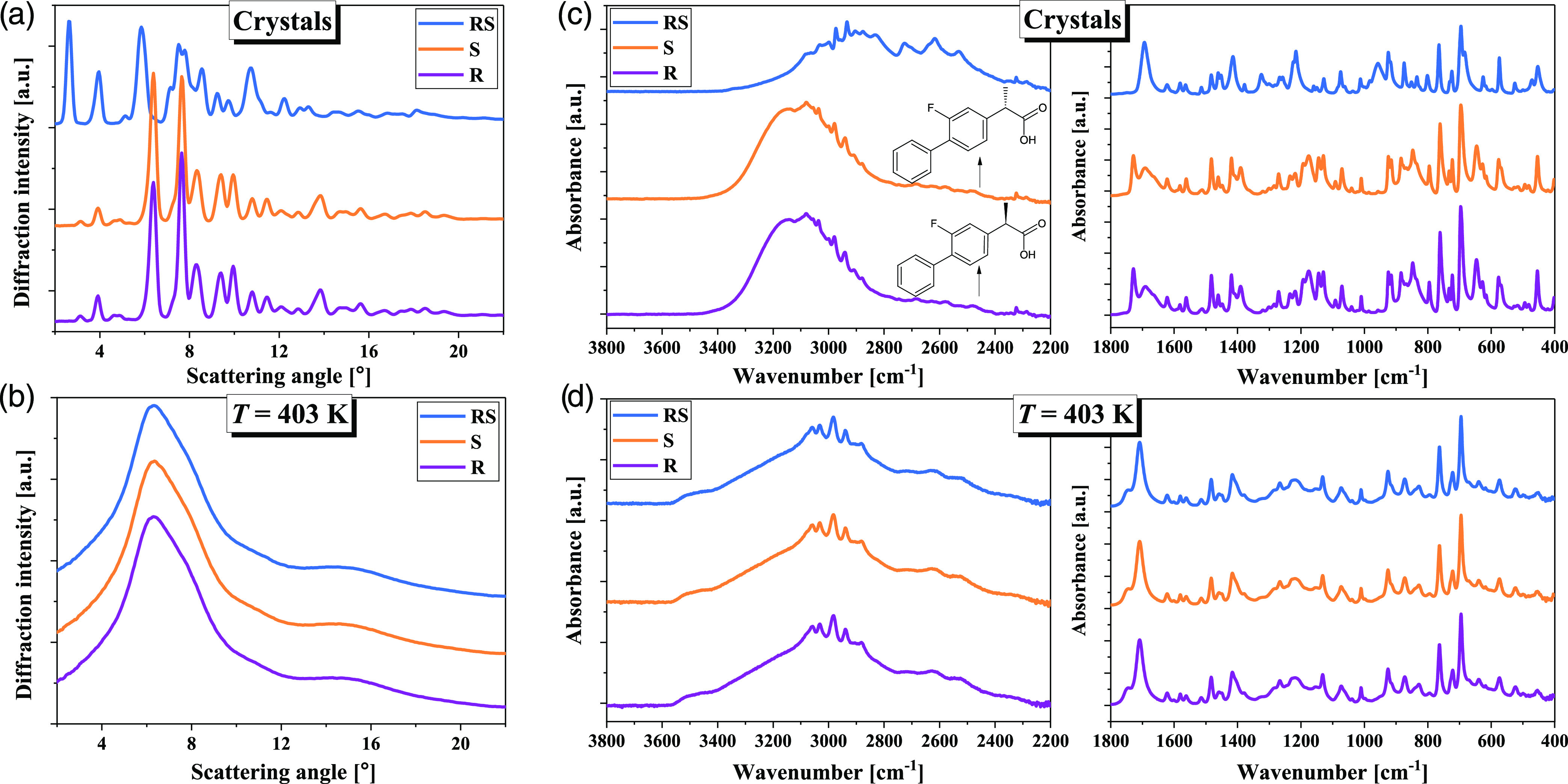
XRD patterns of racemic FLP and both (*R* and *S*) enantiomers in the (a) crystalline (*T* = 293 K) and (b) molten (*T* = 403 K) states. Panels (c, d) show the corresponding FTIR spectra of the same phases in the wavenumber region of 3800–400 cm^–1^. In the insets of panel (c), the structures of *R*- and *S*-FLP are presented.

The first stage of research was to characterize the atomic structure of the examined systems. According to previous studies, three polymorphic forms (I, II, and III) of *RS*-FLP have been reported. However, only the crystal structures of forms I and III have been solved.^64−66^ In both polymorphs, the dimerization of enantiomers through the carboxylic acid group and a similar molecular conformation were recognized, except that in form III, the position of the F atom is disordered over two positions related by a 180° rotation of the F-substituted ring. Based on the diffraction pattern presented in Figure 1a, it was possible to identify the polymorphic form of crystalline racemic FLP as form I. It is characterized by triclinic *P*1 space group and unit cell parameters: 5.8164(2), 9.3136(4), 12.7480(5)Å, 73.136(4), 83.117(3), 72.982(4)° and is the thermodynamically stable form under ambient condition, and hence used in the clinic. In turn, the crystal structure of *R*- and *S*- enantiomers at the same conditions was identical but different from that of racemic FLP, which can be deduced from the following XRD patterns in Figure 1a. This crystal structure has also been reported for the *R*-isomer—as orthorhombic *P*212121 space group and unit cell lengths: 5.6535(3), 13.2097, 16.0709 (5)Å.^67^ It was shown that in such crystal lattice, FLP molecules interacting via H-bonding create chains along the *a*-axis.^67,68^ One can assume that an analogous structural motif occurs for the crystalline sample of the *S*-isomer based on a similar diffraction pattern. Thus, the main difference between the crystal structures of racemic mixture and *R*-, *S*-FLP may be related to the spatial organization of molecules and their interaction through H-bonds with neighboring molecules. In the case of the liquid structure upon melting, the diffraction patterns presented in Figure 1b did not exhibit apparent variations between the chiral and racemic forms.

The different spatial arrangement of the molecules in the crystalline lattice of racemate and pure enantiomers is also well captured in infrared spectra measured in a wide wavenumber range of 3800–400 cm^–1^ (please see Figure 1c). As can be seen, the spectra show the most significant differences in their band contours in the regions corresponding to the vibrations of the groups involved in the association process, i.e., stretching vibration of the H-bonded (HB) hydroxyl (ν_O–H_ 3500–3000 cm^–1^), carbonyl (ν_C=O_ 1800–1640 cm^–1^), carboxylic (ν_C–O_ 1280–1240 cm^–1^), and aliphatic (ν_C–H_ 3000–2400 cm^–1^) moieties as well as out-of-plane deformation of O–H group (γ_O–H_ 980–930 cm^–1^).^69^ It should also be noted that the IR spectrum of racemic FLP exhibits a typical dimeric structure of the ν_O–H_ band consisting of the shorter-wave branch (3500–2750 cm^–1^) of higher intensity and the longer-wave component (2750–2200 cm^–1^) of lower intensity. In contrast, the ν_O–H_ band of *R*-FLP is essentially characterized by a single broad signal, shifted toward higher frequencies relative to *RS*-FLP. Moreover, in a lower wavenumber range of the *RS*-FLP spectrum, an intense and sharp peak at 1694 cm^–1^ due to the stretching vibration of the carbonyl group (ν_C=O_) is observed. On the other hand, the ν_C=O_ band of *R*- and *S*-FLP has a doublet structure consisting of two components (1728 and 1691 cm^–1^). The peak at 1258 cm^–1^ in the infrared spectrum of the racemic FLP is assignable to the C–O stretching and C–O–H deformation vibrations, while for *R*- and *S*-FLP, no signal at this frequency occurs. Instead of that, a peak at 1270 cm^–1^ is detected. A broad, medium-intensity band due to out-of-plane O–H deformation in the *RS*-FLP spectrum can be observed at 957 cm^–1^. In the case of both enantiomers, this peak has a very weak intensity.

As can be seen in Figure 1d, all of these spectral differences related to various spatial arrangements of the molecules in pure enantiomers and the racemate disappear after the sample’s melting, and the spectra of the three examined systems are identical. Moreover, one can postulate that melting of *R*- or *S*- FLP destroys the chain-like arrangement of the molecules as the spectrum of liquid samples closely resembles that of the crystalline racemic FLP (Figure S1 in the Supporting Information). Thus, the broad structure and shape of the ν_O–H_ band after melting are maintained, and the positions of the peaks are similar to those in the racemic FLP crystals. Only the ν_C=O_ band is slightly shifted to the higher wavenumbers (1708 cm^–1^) for molten samples relative to that in the crystalline *RS*-FLP (1694 cm^–1^). This fact may suggest that the HB dimers, with a relatively similar geometry to those in the crystal lattice, exist in the liquid forms of studied *R*-, *S*-, and *RS*-FLP.

**Figure 2:**
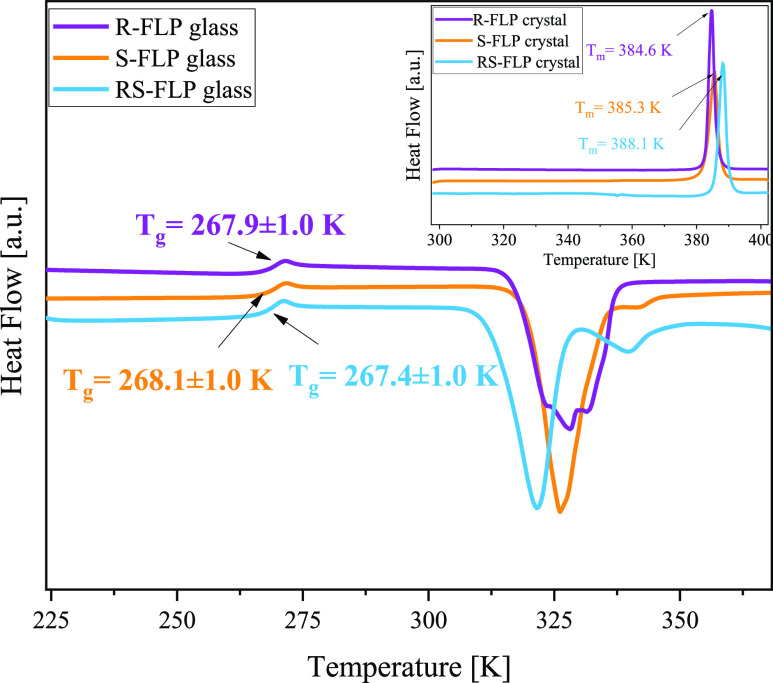
DSC thermograms obtained during heating of the crystalline (inset) and glassy (main panel) *R*-, *S*, and *RS*-FLP at a rate of 10 K/min. *T*_g_ values were determined as the midpoint of the heat capacity increment, while *T*_m_ values were obtained from the maximum of the endothermic peaks.

To verify the eventual differences or similarities in the phase transitions and thermal properties of the crystal and liquid samples, calorimetric measurements were performed. In Figure 2, DSC curves recorded on heating the crystalline (inset) and glassy (main panel) samples at the rate of 10 K/min are presented. As can be seen in the inset, there is one strong endothermic process at *T* = 384.6 K and *T* = 385.3 K for *R*- and *S*-enantiomer, respectively, as well as at *T* = 388.1 K for a racemate, related to the melting of the crystalline samples. It should be mentioned that the obtained values of the melting temperature (*T*_m_) stay in good agreement with those published in the literature (*T*_m_ = 385 K for *S-*FLP^29^ and *T*_m_ = 388 K for *RS*-FLP^70^). Further cooling without any sign of crystallization, followed by heating of the vitrified samples, reveals the presence of one visible glass transition as well as an exothermic peak at around 320 K (a racemate) or 325 K (both enantiomers), corresponding to the cold crystallization. Importantly, the glass-transition temperatures (*T*_g_) of all examined samples are very similar (*T*_g_ = 267.9 ± 1.0, 268.1 ± 1.0, and 267.4 ± 1.0 K, for *R-*, *S-*, and *RS*-FLP, respectively). Moreover, the heat capacity jumps at the *T*_g_ are close to each other (Δ*C*_p_ = 0.373, 0.357, and 0.375 J g^1–^ K^–1^, for *R-*, *S-*, and *RS*-FLP, respectively).

**Figure 3:**
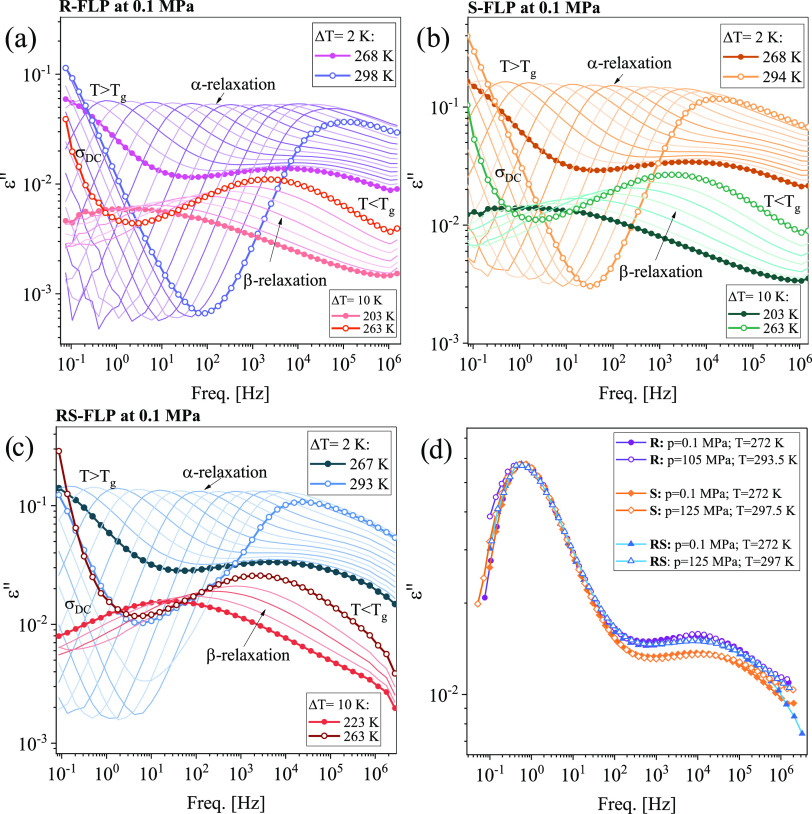
Dielectric loss spectra measured for *R*-FLP (a), *S*-FLP (b), and *RS*-FLP (c) at ambient pressure in a wide temperature range, above and below the *T*_g_. A comparison of dielectric spectra obtained at different thermodynamic conditions (*T*, *p)*, close to *T*_g_, is shown for *R*, *S*, and *RS*-FLP (d). They were normalized with respect to the maximum of dielectric loss (ε″_max_).

Subsequently, having the results of previous investigations in mind, the molecular dynamics of *R*-, *S*-, and *RS*-FLP at ambient and elevated pressure (*p*) conditions were followed using the BDS technique. Figure 3a–c presents dielectric loss spectra of all samples collected at *p* = 0.1 MPa and in a wide range of temperatures (*T*), both above and below the *T*_g_. In the supercooled liquid phase (*T > T*_g_), two characteristic processes can be identified in the spectra of pure FLP enantiomers and the racemic mixture. The first one, whose source is charge transport of ionic impurities, is the dc-conductivity (σ_dc_), while the second one, located at higher frequencies (*f*), is a structural (α)-relaxation related to cooperative motions of all molecules in the examined samples. As illustrated, both processes shift toward lower values of *f* with a decreasing *T*. In turn, in the glassy state (*T < T*_g_), one well-visible secondary relaxation, labeled β, is observed in dielectric loss spectra for all investigated systems.

To determine relaxation times of the structural (α) process, dielectric data collected at *p* = 0.1 MPa and *T* > *T*_g_ were fitted using the Havriliak–Negami (HN) function^71^
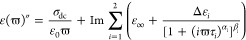
2where σ_dc_ is the dc-conductivity, ε_0_ is the vacuum permittivity, ω̅ is an angular frequency (ω̅ = 2π*f*), ε_∞_ is the high-frequency limit permittivity, Δε is the dielectric relaxation strength, τ is the HN relaxation times, and α and β are the shape parameters that represent the symmetric and asymmetric broadening of given relaxation peaks. Then, using the expression given in a book by Kremer and Schönhals,^43^
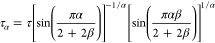
3τ_α_ have been recalculated from τ. To describe the *T*-dependencies of τ_α_ (see Figure S2 in the Supporting Information) at 0.1 MPa, the Vogel–Fulcher–Tammann (VFT) equation^72−74^ was applied. Subsequently, based on the VFT fits, the *T*_g_ (defined as a *T* at which τ_α_ = 100 s) for pure enantiomers and the racemic mixture was estimated. It should be noted that the obtained values (*T*_g_ = 263.1 ± 1.0, 263.4 ± 1.0, and 261.9 ± 1.0 K for *R*-, *S*-, and *RS*-FLP, respectively) do not differ significantly from each other and are only a few Kelvins lower when compared to those estimated from the DSC technique (see Figure 2). At first sight, one can get the impression that the relaxation time at the vitrification point is different when we analyze dielectric and calorimetric data. However, it is a common observation reported in the literature for many systems that is predominantly related to a difference in the heating rates applied during both experiments.

**Figure 4:**
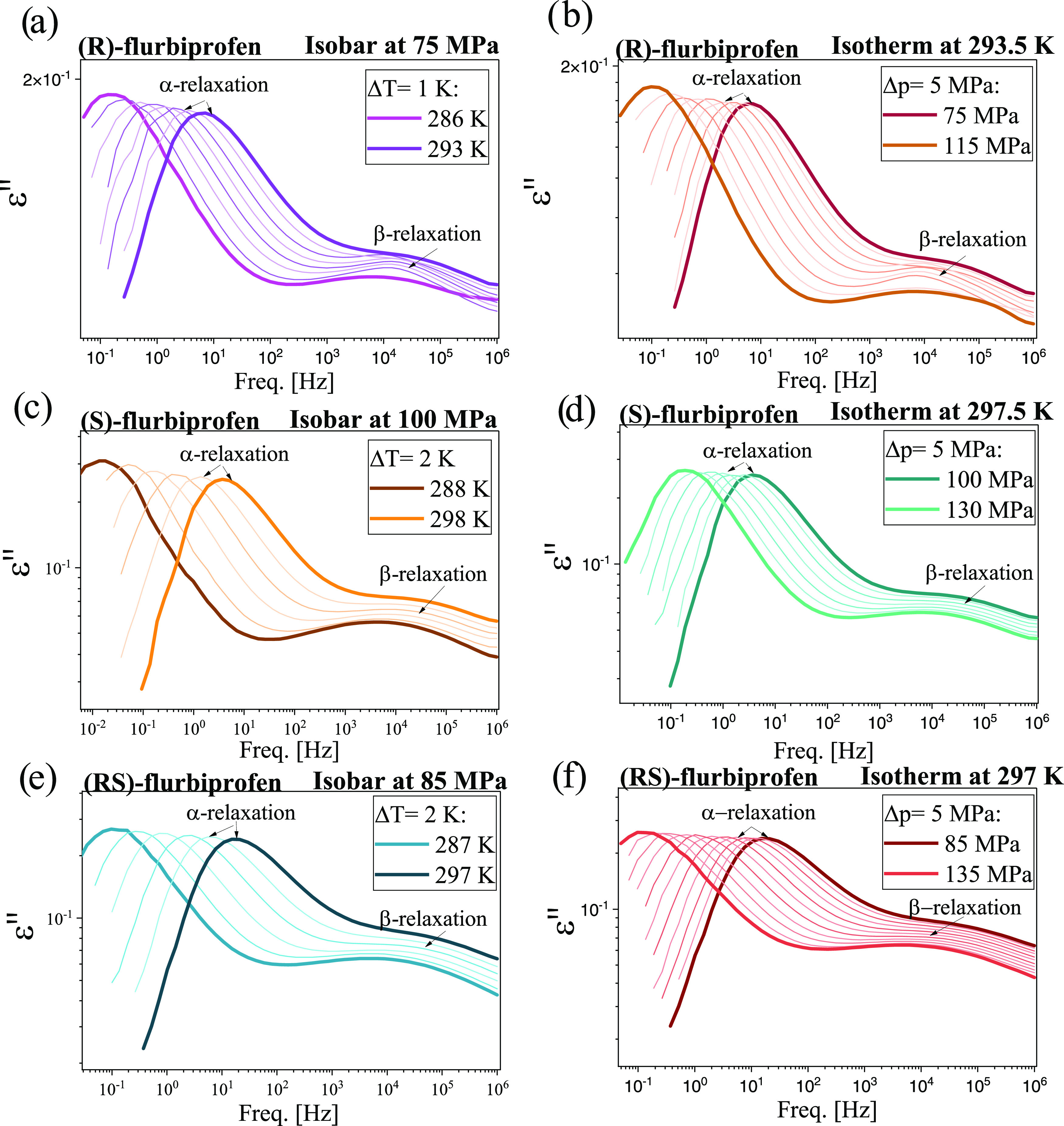
Representative dielectric loss spectra measured for *R*-, *S*-, and *RS*-FLP at isobaric (panels (a), (c), (e)) and isothermal (panels (b), (d), (f)) conditions.

Additionally, extensive high-pressure BDS investigations under isobaric and isothermal conditions were carried out. In Figure 4, representative dielectric loss spectra collected for pure enantiomers and a racemic FLP at constant *p* and various *T* > *T*_g_ (panels a,c,e), as well as at constant *T* and indicated *p* < *p*_g_, where *p*_g_ is a glass-transition pressure (panels b,d,f), are shown. Similar to the ambient pressure studies, except for the dc-conductivity, a structural (α)-relaxation peak (whose maximum moves toward lower *f* with decreasing *T* or increasing *p*), as well as one secondary (β)-relaxation, are observed in the spectra of all examined substances.

In Figure 3d, the normalized dielectric spectra measured for *R*-, *S*-FLP, and *RS*-FLP at ambient and high *p*, and *T* close to *T*_g_ (the maxima of α-peaks near 1 Hz) were compared. As can be seen, for all investigated samples, the position of α- and secondary (β)-relaxation peaks is the same at various combinations of *T* and *p*, which means the fulfillment of the isochronal α- and β-superpositioning (the rule, whose confirmation has been reported for many HB systems^75−78^). However, it can also be observed that for both FLP enantiomers, the left-hand side of α-dispersion broadens with compression, indicating the violation of the so-called temperature pressure superpositioning (TPS).^79^ This effect is more pronounced for the *R*-isomer. Surprisingly, in the case of the racemic FLP, the TPS is satisfied (the width of the structural relaxation remains unchanged under various *T* and *p* conditions).

**Figure 5:**
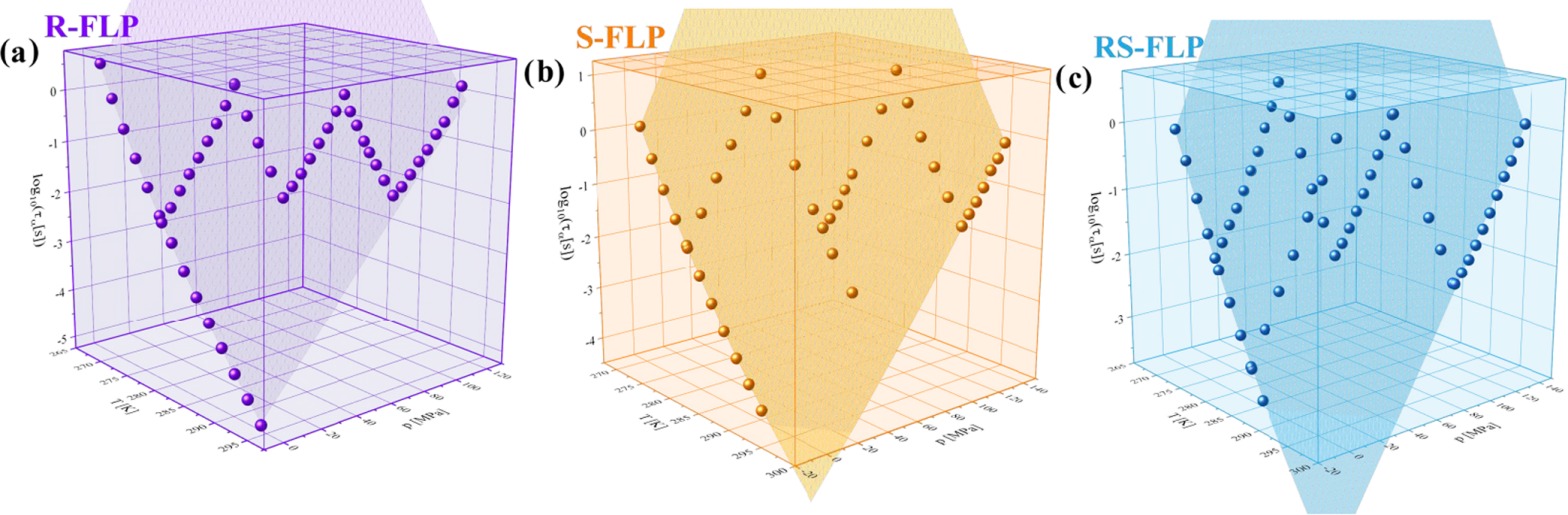
Structural relaxation times of *R*- (a), *S*- (b), and *RS*-FLP (c) plotted versus temperature (*T*) and pressure (*p*). Purple, orange, and blue areas represent surface fits to the modified Avramov eq (eq 4).

As the further part of the in-depth analysis of the molecular dynamics of *R*-, *S*-FLP, and *RS*-FLP, BDS data shown in Figure 4 were analyzed using the superposition of two HN functions (eq 2). Then, the obtained τ_α_ (also those determined from ambient-*p* experiments) were plotted as a function of *T* and *p* in Figure 5 and then analyzed with the use of the modified Avramov equation^80^
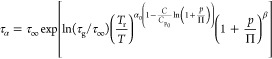
4where τ_∞_ is a relaxation time at extremely high *T*, τ_g_ = τ(*T*_g_), *T*_*r*_ is a reference temperature lying close to the *T*_g_, *C*_p_0__ is a specific heat capacity, *C* is an additional adjustable parameter, and Π is a constant with the dimension of pressure, while α_0_ and β are exponential parameters defined as
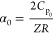
5
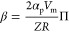
6where *Z* is the degeneracy of the system, α_p_ is a volume expansion coefficient at 0.1 MPa, and *V*_m_ is a molar volume. In Table S1 in the Supporting Information, the parameters of eq 4 determined from the global numerical fitting procedures (Figure 5) are presented.

**Figure 6:**
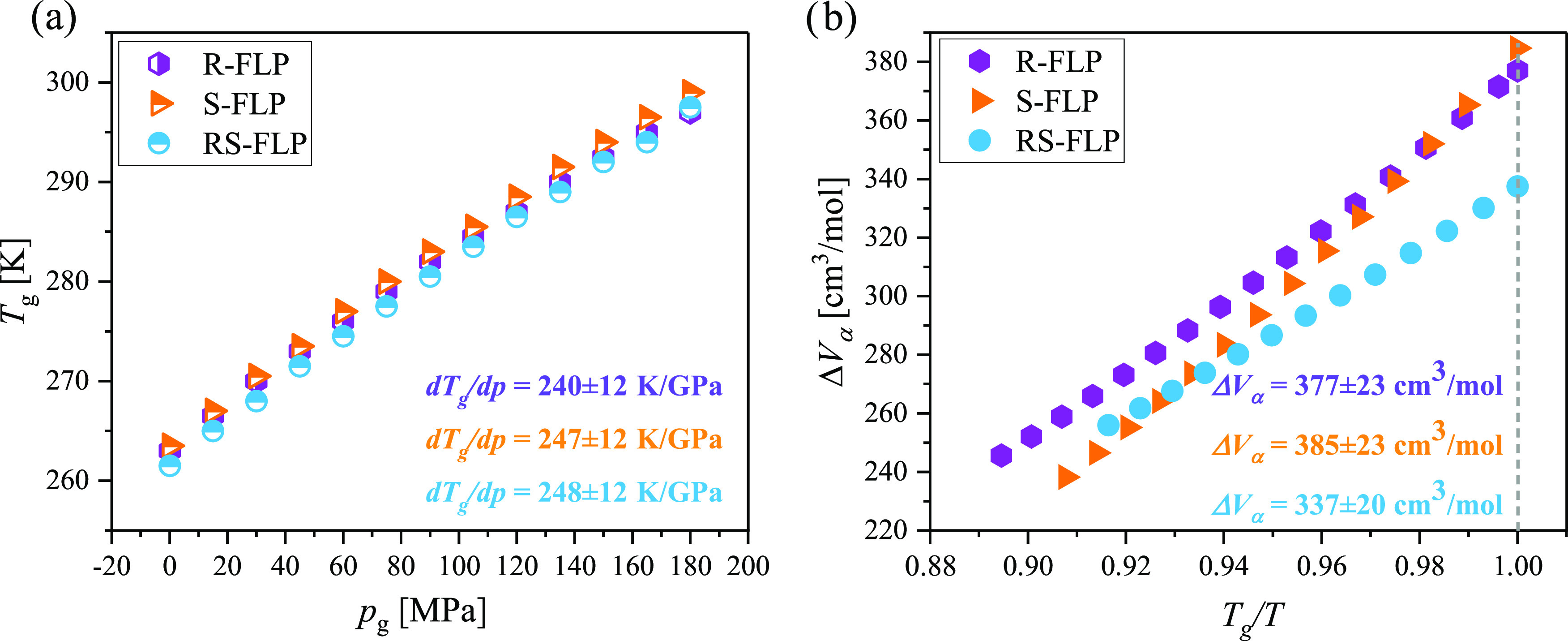
*T*_g_ plotted as a function of *p*_g_ (a). Dependence of the activation volume for the α-process (Δ*V*_α_) versus *T*_g_/*T* (b).

Subsequently, two quantities, which define the sensitivity of the α-process to compression, i.e., the pressure coefficient of the glass-transition temperature (d*T*_g_/d*p*) and the activation volume (Δ*V*_α_), were estimated. To determine the former one, *T*_g_s obtained for both enantiomers and the racemic FLP by applying the following expression proposed by Avramov^81^
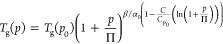
7with the same values of the parameters *C*_p_0__, Π, β, and α_0_ as those appearing in eq 4, were presented as a function of pressure (Figure 6a). Interestingly, d*T*_g_/d*p*s obtained from this plot are almost the same for all examined systems (d*T*_g_/d*p*^*R*-FLP^ = 240 ± 12 K/GPa, d*T*_g_/d*p*^*S*-FLP^ = 247 ± 12, K/GPa, and d*T*_g_/d*p*^*RS*-FLP^ = 248 ± 12 K/GPa) and signify a high sensitivity of the structural relaxation to pressure. Note that somewhat lower values of this parameter have been previously reported for the two other APIs from the profens’ group—ibuprofen (d*T*_g_/d*p* = 195 K/GPa)^82^ and ketoprofen (d*T*_g_/d*p* = 200 K/GPa).^26^

On the other hand, Δ*V*_α_ for all examined systems (FLP enantiomers and the racemate), usually defined as^45^
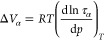
8was determined at different *T* directly from the surface fit of the modified Avramov approach. The plot presenting Δ*V*_α_ versus *T*_g_/*T* is shown in Figure 6b. Notably, the values of this parameter calculated for pure enantiomers at *T*_g_ (*T*_g_/*T* = 1) were very close and equal to 377 ± 23 and 385 ± 23 cm^3^/mol for *R*- and *S*-FLP. In turn, surprisingly, Δ*V*_α_ obtained for the racemic mixture is clearly lower (Δ*V*_α_ = 337 ± 20 cm^3^/mol) in comparison to the values obtained for both isomers. This unexpected variation in Δ*V*_α_ between pure FLP enantiomers and racemate indicates that the local spatial arrangement of the molecules in the liquid state of these systems may not be the same. Hence, some memory of the crystalline molecular arrangement, although not detectable with the experimental techniques applied in our studies, survived upon melting and in the vicinity of the *T*_g_. To confirm this hypothesis, in-depth molecular dynamics (MD) simulations in the supercooled liquid state of *S*- and *RS*-FLP were performed.

The final simulations (after the initial equilibration) of FLP systems were taken at ambient pressure and a temperature of 293 K. The first analysis was related to the clusterization effect by H-bonds. It was found that hydrogen-bond-based clusters are more abundant in the enantiomeric system than in the racemate. Moreover, the difference in the total clusters, although noticeable, is relatively small. As presented in the histogram in Figure S3 in the Supporting Information, there is only a 0.5% difference in the overall amount of HB clusters. Nevertheless, a more significant difference can be found in their distribution. In the case of the *S*-isomer, there is a higher population of low-molecular-weight clusters (dimer to tetramer). The percentage of low-molecular HB clusters (2–4 molecules) equals 56% in the enantiomer and 44% in the racemate.

**Figure 7:**
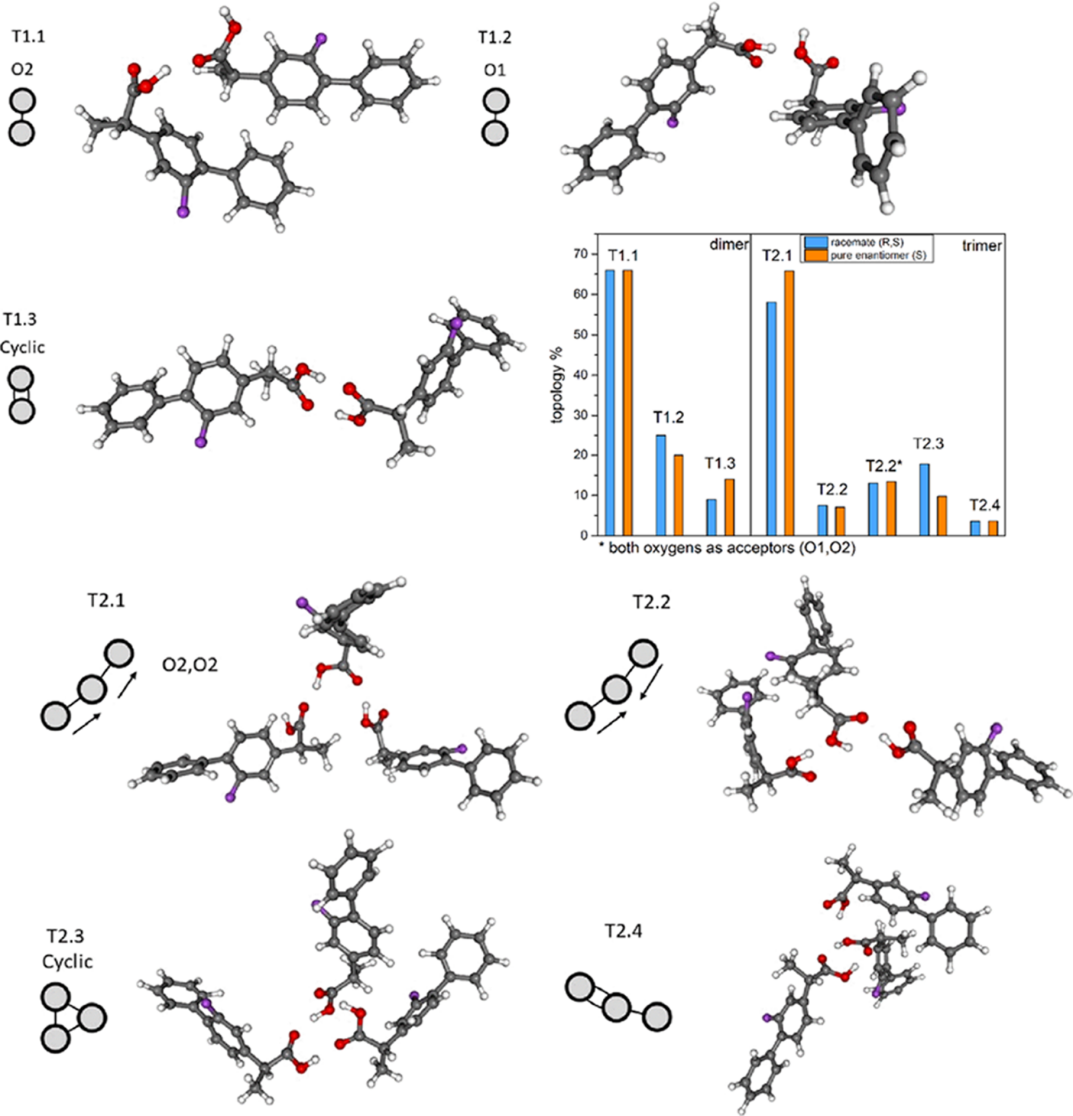
Topology of the most abundant H-bonds between two flurbiprofen molecules that can be found in the supercooled systems. T1.1, T1.2, and T1.3 are the most abundant dimer topologies, while T2.1–T2.4 are the most abundant trimer topologies.

In both systems, there is also a small population of cyclic dimers and trimers. As illustrated in Figure 7, showing the detailed topology of H-bonds, cyclic dimers constitute 13 and 9% of all dimers in the *S*-isomer and *RS*-racemate, respectively. In the case of trimers, more abundant cyclic structures occur in the racemic mixture (18% of all trimers in *RS* versus 9% of all trimers in the *S*-enantiomer).

**Figure 8:**
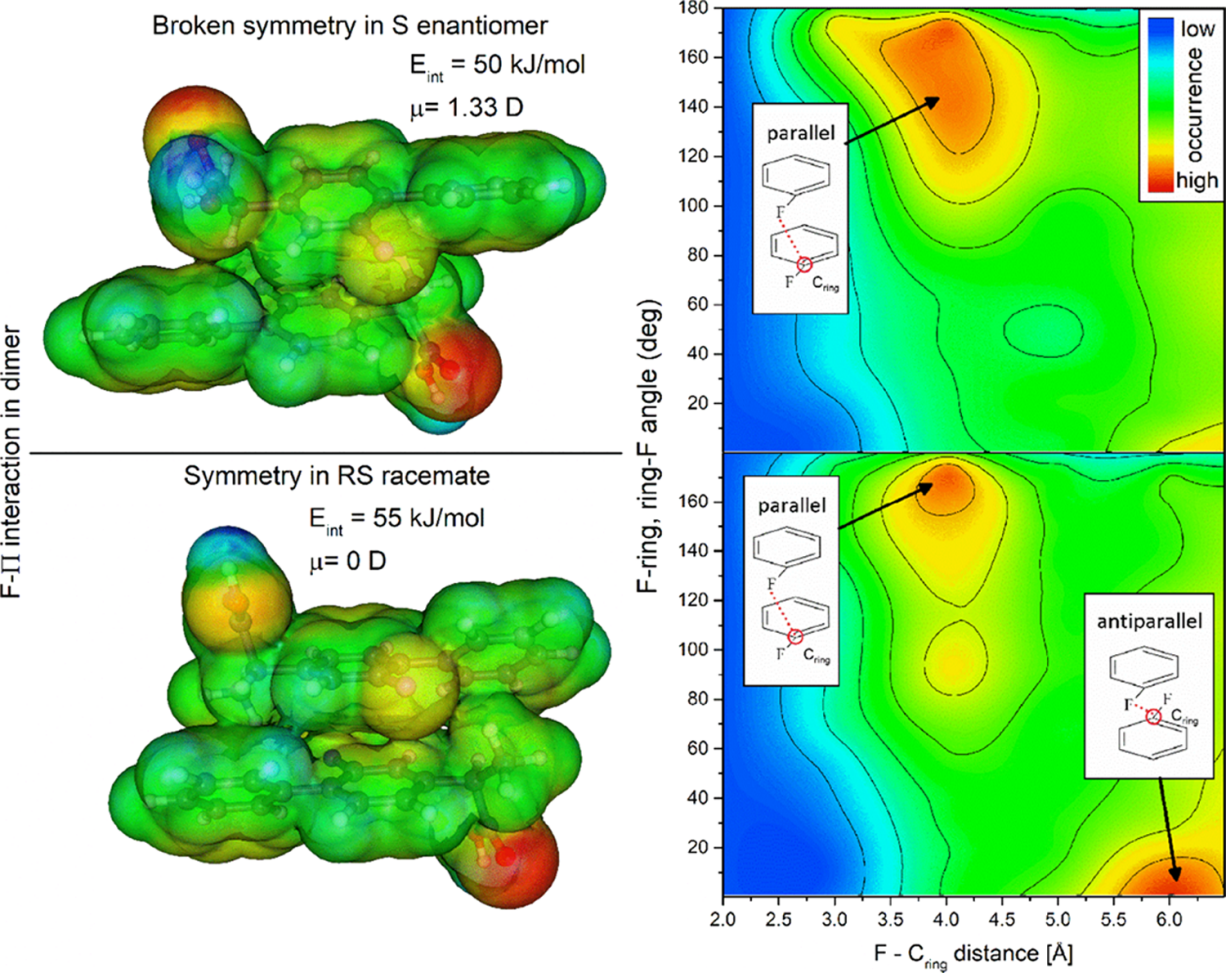
Visualization of the F–Π interactions in *RS*- and *S*-FLP systems. In the left panel, the geometry and electrostatic potential of R-S and S–S non-HB dimers are presented. In the right panel, RDF vs ADF 2D maps are illustrated to show the abundance of parallel and antiparallel Π-stacking formations.

Based on the data obtained from MD simulations, one can suppose that the higher activation volume obtained from dielectric investigations for the pure enantiomers with regard to the racemate is a manifestation of the difference in clusterization pattern and a higher population of the di-, tri-, and tetramers in the former systems. As a consequence of that, there is a larger average cluster size in the enantiomer. Having that in mind, one can ask the question, what is a driving force underlying the formation of extensive clusters in a pure isomer? To address this question, the OH···O hydrogen bond energy between *S*–*S* and *R*–*S* molecules was calculated. It was found that the interaction energies (*E*_int_) between *S*–*S* and *R*–*S* molecules are similar. The obtained value was equal to 105 and 100 kJ/mol for the former and latter systems, respectively. The calculated *E*_int_ is unusually high if only a single OH···O hydrogen bond is considered. It became clear that the conformation of linearly H-bonded two FLP molecules has additional Π-stacking aromatic rings. Moreover, due to the fluorine (F) atom connected with the ring, the strength of such interaction can be higher because of the particular case of Π-stacking, i.e., F–Π interaction. As this was found to be a concurrent interaction with the H-bonds, MD simulations were analyzed to show possible differences in Π-stacking conformations. It was achieved by plotting 2D maps of the radial distribution function (RDF) of ring atoms versus the angular distribution function (ADF) of ring plane vectors. In the right panel of Figure 8, the 2D map of intermolecular RDF between F and C_ring_ (where C_ring_ is the ring atom connected with the F atom) versus the ADF between F–C_ring_ and F–C_ring_ vectors, indicating significant differences in the Π-stacked structures, are presented. As can be observed, there are two possible Π-stacking formations. The first one is when aromatic rings with fluorine atoms are parallel to each other. In this scenario, the angle on the map is close to 180°, and the distance between the F and C_ring_ atoms is approximately equal to 4 Å. The second possible arrangement is when both rings are antiparallel to each other. The angle on the map is then close to 0°, and the distance between F and C_ring_ is approximately equal to 6 Å. It is visible that in the case of the *RS*-racemate, both arrangements, i.e., ordered parallel and antiparallel structures, are abundant. For the *S*-enantiomer, only parallel structures are detected, but in this case, there is a broader angle distribution, which means that in these structures, rings are not so well parallel aligned. This finding corresponds very well with the crystalline patterns of the *R*-*S* and *S–S* structures. The antiparallel alignment found in the racemate is the precursor of the F–Π interacting conformation, which can be easily found in the unit cell of triclinic *R*-*S* crystals (see Figure S4 in the Supporting Information for details). In the left panel of Figure 8, two structures of *R*-*S* and *S*-*S* dimers bonded exclusively by Π-stacking forces are shown. These structures were optimized by the density functional theory (DFT) method and presented as electron density with mapped electrostatic potential on their surface. The *R*-*S* unit is the same as that found in the crystal cell. The *S*-*S* unit was optimized from the initial *R*-*S* structure after switching the chirality of the asymmetric carbon in one molecule. As one can see, the *R*-*S* structure is perfectly symmetric, which is manifested in the zero dipole moment (μ = 0 D). In the case of the *S*-*S* conformation, there are visible distortions in the charge distribution and its symmetry. The dipole moment of *S*-*S* molecules is equal to 1.33 D. The F−Π stacking energy is equal to 55 and 50 kJ/mol in *R-S* and *S*-*S* systems, respectively. Therefore, in the case of *S*-FLP, as the enantiomeric molecules are unable to form symmetric F−Π structures, the system is dominated more by H-bonds, which further leads to the different crystalline structure (an orthorhombic structure in the case of the *S*-enantiomer). Moreover, as it was found that the energy of F−Π interaction is comparable to OH···O hydrogen bond, the bonding energy of linear dimer, where there are contributions of both types of interactions, is comparable to the bonding energy of cyclic dimer. This is the reason why both supercooled systems are dominated by the linear HB clusters and not seemingly more stable cyclic dimers.

## 4 Conclusions

To summarize, the studies indicated much different molecular organization via H-bonds in pure enantiomers (long chains) and racemate (cyclic dimers) in the crystalline samples of flurbiprofen. However, once the crystalline lattice is destroyed, these differences vanish, as can be deduced from the experimental data. According to the collected diffractograms, thermograms, infrared, and dielectric spectra, enantiomers have properties identical with those of the racemate. However, a deeper analysis of dielectric data revealed an unexpected variation in the activation volume, which may be related to changes in the local molecular arrangement of these compounds. In fact, MD simulations confirmed this supposition, indicating a higher population of smaller supramolecular clusters in *S*-FLP with regard to the *RS*-FLP. Finally, it was also found that there are very specific and enormously strong F−Π interactions in the studied systems that may control local molecular arrangement in the crystalline and supercooled samples. The presented data contribute to a better understanding of the correlation among the structure, intermolecular interactions, and basic physical properties of the enantiomers and racemates.

## Supporting Information Available

Comparison of FTIR spectra of crystalline and molten *RS*-FLP, relaxation map for FLP enantiomers, and a racemate obtained from the analysis of ambient pressure BDS data, parameters of the modified Avramov eq (eq 4), as well as additional outcomes of MD simulations and DFT calculations (PDF)

## References

[ref1] Naaman R.; Paltiel Y.; Waldeck D. H. Chiral Induced Spin Selectivity and Its Implications for Biological Functions. Annu. Rev. Biophys. 2022, 51, 99–114. 10.1146/annurev-biophys-083021-070400. 34932912

[ref2] Zhao X.; Zang S. Q.; Chen X. Stereospecific Interactions between Chiral Inorganic Nanomaterials and Biological Systems. Chem. Soc. Rev. 2020, 49, 2481–2503. 10.1039/D0CS00093K. 32176233

[ref3] Michaeli K.; Naaman R. Origin of Spin-Dependent Tunneling Through Chiral Molecules. J. Phys. Chem. C 2019, 123, 17043–17048. 10.1021/acs.jpcc.9b05020.

[ref4] Naaman R.; Paltiel Y.; Waldeck D. H. Chiral Molecules and the Electron Spin. Nat. Rev. Chem. 2019, 3, 250–260. 10.1038/s41570-019-0087-1.

[ref5] Greenfield J. L.; Wade J.; Brandt J. R.; Shi X.; Penfold T. J.; Fuchter M. J. Pathways to Increase the Dissymmetry in the Interaction of Chiral Light and Chiral Molecules. Chem. Sci. 2021, 12, 8589–8602. 10.1039/D1SC02335G. 34257860 PMC8246297

[ref6] Muñoz J.; González-Campo A.; Riba-Moliner M.; Baeza M.; Mas-Torrent M. Chiral Magnetic-Nanobiofluids for Rapid Electrochemical Screening of Enantiomers at a Magneto Nanocomposite Graphene-Paste Electrode. Biosens. Bioelectron. 2018, 105, 95–102. 10.1016/j.bios.2018.01.024. 29412951

[ref7] Fernández-García J. M.; Evans P. J.; Filippone S.; Herranz M. Á.; Martín N. Chiral Molecular Carbon Nanostructures. Acc. Chem. Res. 2019, 52, 1565–1574. 10.1021/acs.accounts.9b00144. 31181912

[ref8] Abram M.; Jakubiec M.; Kamiński K. Chirality as an Important Factor for the Development of New Antiepileptic Drugs. ChemMedChem 2019, 14, 1744–1761. 10.1002/cmdc.201900367. 31476107

[ref9] Ribeiro A. R.; Castro P. M. L.; Tiritan M. E. Chiral Pharmaceuticals in the Environment. Environ. Chem. Lett. 2012, 10, 239–253. 10.1007/s10311-011-0352-0.

[ref10] Higuchi A.; Tamai M.; Ko Y.-A.; Tagawa Y.-I.; Wu Y.-H.; Freeman B. D.; Bing J.-T.; Chang Y.; Ling Q.-D. Polymeric Membranes for Chiral Separation of Pharmaceuticals and Chemicals. Polym. Rev. 2010, 50, 113–143. 10.1080/15583721003698853.

[ref11] Coquerel G.; Tamura R. “Enantiomeric Disorder” Pharmaceutically Oriented. Disord. Pharm. Mater. 2016, 135–160. 10.1002/9783527652693.ch5.

[ref12] Tokunaga E.; Yamamoto T.; Ito E.; Shibata N. Understanding the Thalidomide Chirality in Biological Processes by the Self-Disproportionation of Enantiomers. Sci. Rep. 2018, 8, 17131 10.1038/s41598-018-35457-6. 30459439 PMC6244226

[ref13] Blaschke G.; Kraft H. P.; Fickentscher K.; Köhler F. Chromatographic separation of racemic thalidomide and teratogenic activity of its enantiomers (author’s transl). Arzneimittel-forschung 1979, 29, 1640–1642. 583234

[ref14] Rulcova A.; Prokopova I.; Krausova L.; Bitman M.; Vrzal R.; Dvorak Z.; Blahos J.; Pavek P. Stereoselective Interactions of Warfarin Enantiomers with the Pregnane X Nuclear Receptor in Gene Regulation of Major Drug-Metabolizing Cytochrome P450 Enzymes. J. Thromb. Haemostasis 2010, 8, 2708–2717. 10.1111/j.1538-7836.2010.04036.x. 20735727

[ref15] Miyazaki K.; Kaiho F.; Inagaki A.; Dohi M.; Hazemoto N.; Haga M.; Hara H.; Kato Y. Enantiomeric Difference in Percutaneous Penetration of Propranolol Through Rat Excised Skin. Chem. Pharm. Bull. 1992, 40, 1075–1076. 10.1248/cpb.40.1075. 1525940

[ref16] Kamalzadeh Z.; Babanezhad E.; Ghaffari S.; Mohseni Ezhiyeh A.; Mohammadnejad M.; Naghibfar M.; Bararjanian M.; Attar H. Determination of Bortezomib in API Samples Using HPLC: Assessment of Enantiomeric and Diastereomeric Impurities. J. Chromatogr. Sci. 2017, 55, 697–705. 10.1093/chromsci/bmx023. 28369337

[ref17] Kakumanu V. K.; Arora V.; Bansal A. K. Investigation on Physicochemical and Biological Differences of Cefpodoxime Proxetil Enantiomers. Eur. J. Pharm. Biopharm. 2006, 64, 255–259. 10.1016/j.ejpb.2006.05.001. 16797950

[ref18] Waldeck B. Biological Significance of the Enantiomeric Purity of Drugs. Chirality 1993, 5, 350–355. 10.1002/chir.530050514. 8398592

[ref19] Younes A. A.; Ates H.; Mangelings D.; Heyden Y. V. A Separation Strategy Combining Three HPLC Modes and Polysaccharide-Based Chiral Stationary Phases. J. Pharm. Biomed. Anal. 2013, 75, 74–85. 10.1016/j.jpba.2012.11.019. 23312387

[ref20] Ward T. J.; Ward K. D. Chiral Separations: A Review of Current Topics and Trends. Anal. Chem. 2012, 84, 626–635. 10.1021/ac202892w. 22066781

[ref21] Younes A. A.; Mangelings D.; Heyden Y. V. Chiral Separations in Normal-Phase Liquid Chromatography: Enantioselectivity of Recently Commercialized Polysaccharide-Based Selectors. Part II. Optimization of Enantioselectivity. J. Pharm. Biomed. Anal. 2011, 56, 521–537. 10.1016/j.jpba.2011.06.011. 21757311

[ref22] Thunberg L.; Hashemi J.; Andersson S. Comparative Study of Coated and Immobilized Polysaccharide-Based Chiral Stationary Phases and Their Applicability in the Resolution of Enantiomers. J. Chromatogr. B 2008, 875, 72–80. 10.1016/j.jchromb.2008.07.044. 18723406

[ref23] Phadnis N. V.; Suryanarayanan R. Simultaneous Quantification of an Enantiomer and the Racemic Compound of Ibuprofen by X-Ray Powder Diffractometry. Pharm. Res. 1997, 14, 1176–1180. 10.1023/A:1012198605891. 9327444

[ref24] Yuan X.; Li X.; Guo P.; Xiong Z.; Zhao L. Simultaneous Enantiomeric Analysis of Chiral Non-Steroidal Anti-Inflammatory Drugs in Water, River Sediment, and Sludge Using Chiral Liquid Chromatography-Tandem Mass Spectrometry. Anal. Methods 2018, 10, 4404–4413. 10.1039/C8AY01417E.

[ref25] Caldwell J.; Hutt A. J.; Fournel-Gigleux S. The Metabolic Chiral Inversion and Dispositional Enantioselectivity of the 2-Arylpropionic Acids and Their Biological Consequences. Biochem. Pharmacol. 1988, 37, 105–114. 10.1016/0006-2952(88)90762-9. 3276314

[ref26] Adrjanowicz K.; Kaminski K.; Paluch M.; Niss K. Crystallization Behavior and Relaxation Dynamics of Supercooled S-Ketoprofen and the Racemic Mixture along an Isochrone. Cryst. Growth Des. 2015, 15, 3257–3263. 10.1021/acs.cgd.5b00373.

[ref27] Hachuła B. The Nature of Hydrogen-Bonding Interactions in Nonsteroidal Anti-Inflammatory Drugs Revealed by Polarized IR Spectroscopy. Spectrochim. Acta, Part A 2018, 188, 189–196. 10.1016/j.saa.2017.07.005. 28711781

[ref28] Cao S.; Zhou Y.; Ma Q.; Zhang J.; Wang Z. Experimental and Computational Studies of Enantioseparation of Three Profen Enantiomers with a Focus on Quantification of the Enantiomeric Impurities Present in the Corresponding Enantiopure S-Profen Drugs. J. Chromatogr. A 2022, 1673, 463095 10.1016/j.chroma.2022.463095. 35537349

[ref29] Rodrigues A. C.; Viciosa M. T.; Danède F.; Affouard F.; Correia N. T. Molecular Mobility of Amorphous S-Flurbiprofen: A Dielectric Relaxation Spectroscopy Approach. Mol. Pharmaceutics 2014, 11, 112–130. 10.1021/mp4002188. 24215236

[ref30] Rigoni L.; Venti S.; Bevilacqua M.; Bucci R.; Magrì A. D.; Magrì A. L.; Marini F. Quantification of the Enantiomeric Excess of Two APIs by Means of near Infrared Spectroscopy and Chemometrics. Chemom. Intell. Lab. Syst. 2014, 133, 149–156. 10.1016/j.chemolab.2014.02.004.

[ref31] Hao H.; Wang G.; Sun J. Enantioselective Pharmacokinetics of Ibuprofen and Involved Mechanisms. Drug Metab. Rev. 2005, 37, 215–234. 10.1081/DMR-200047999. 15747501

[ref32] Sonawane H. R.; Bellur N. S.; Kulkarni D. G.; Ayyangar N. R. Photochemical Rearrangement of α-Chloro-Propiophenones to α-Arylpropanoic Acids: Studies on Chirality Transfer and Synthesis of (S)-(+)-Ibuprofen and (S)-(+)-Ketoprofen. Tetrahedron 1994, 50, 1243–1260. 10.1016/S0040-4020(01)80835-8.

[ref33] Caballo C.; Sicilia M. D.; Rubio S. Enantioselective Determination of Representative Profens in Wastewater by a Single-Step Sample Treatment and Chiral Liquid Chromatography–Tandem Mass Spectrometry. Talanta 2015, 134, 325–332. 10.1016/j.talanta.2014.11.016. 25618675

[ref34] Ottou Abe M. T.; Viciosa M. T.; Correia N. T.; Affouard F. Impact of Chirality on Peculiar Ibuprofen Molecular Dynamics: Hydrogen Bonding Organization and Syn vs. Anti Carboxylic Group Conformations. Phys. Chem. Chem. Phys. 2018, 20, 29528–29538. 10.1039/C8CP04837A. 30457612

[ref35] Emel’Yanenko V. N.; Stange P.; Feder-Kubis J.; Verevkin S. P.; Ludwig R. Dissecting Intermolecular Interactions in the Condensed Phase of Ibuprofen and Related Compounds: The Specific Role and Quantification of Hydrogen Bonding and Dispersion Forces. Phys. Chem. Chem. Phys. 2020, 22, 4896–4904. 10.1039/C9CP06641A. 31930249

[ref36] Adrjanowicz K.; Kaminski K.; Tarnacka M.; Szutkowski K.; Popenda L.; Bartkowiak G.; Paluch M. The Effect of Hydrogen Bonding Propensity and Enantiomeric Composition on the Dynamics of Supercooled Ketoprofen - Dielectric, Rheological and NMR Studies. Phys. Chem. Chem. Phys. 2016, 18, 10585–10593. 10.1039/C6CP00578K. 27035123

[ref37] Atawa B.; Couvrat N.; Affouard F.; Correia N. T.; Coquerel G.; Saiter-Fourcin A. Impact of Chirality on the Amorphous State of Conglomerate Forming Systems: A Case Study of N-Acetyl-α-Methylbenzylamine. Phys. Chem. Chem. Phys. 2021, 23, 24282–24293. 10.1039/D1CP03843E. 34672303

[ref38] Roszak K.; Katrusiak A. High-Pressure Crystallization and Thermodynamic Stability Study on the Resolution of High-Density Enantiomers from Low-Density Racemates. Org. Lett. 2023, 25, 37–41. 10.1021/acs.orglett.2c03747. 36598359 PMC9841603

[ref39] Rietveld I. B.; Barrio M.; Tamarit J.-L.; Do B.; Céolin R. Enantiomer Resolution by Pressure Increase: Inferences from Experimental and Topological Results for the Binary Enantiomer System (R)- and (S)-Mandelic Acid. J. Phys. Chem. B 2011, 115, 14698–14703. 10.1021/jp209328d. 22047025

[ref40] Ostrowska K.; Kropidłowska M.; Katrusiak A. High-Pressure Crystallization and Structural Transformations in Compressed R,S-Ibuprofen. Cryst. Growth Des. 2015, 15, 1512–1517. 10.1021/cg5018888.

[ref41] Jacques J.; Collet A.; Wilen S. H. Enantiomers, Racemates, and Resolutions; Wiley: New York, 1981.

[ref42] Koperwas K.; Tu W.; Affouard F.; Adrjanowicz K.; Kaskosz F.; Paluch M. Pressure Dependence of the Crystallization Rate for the S-Enantiomer and a Racemic Mixture of Ibuprofen. Cryst. Growth Des. 2021, 21, 7075–7086. 10.1021/acs.cgd.1c00980. PMC8641391 34880715

[ref43] Kremer F.; Schönhals A. Broadband Dielectric Spectroscopy; Springer: Berlin, 2003.

[ref44] Roland C. M.; Hensel-Bielowka S.; Paluch M.; Casalini R. Supercooled Dynamics of Glass-Forming Liquids and Polymers under Hydrostatic Pressure. Rep. Prog. Phys. 2005, 68, 1405–1478. 10.1088/0034-4885/68/6/R03.

[ref45] Floudas G.; Paluch M.; Ngai K. Molecular Dynamics of Glass-Forming Systems: Effects of Pressure; Springer: Berlin, 2011.

[ref46] Bekker H.; Berendsen H. J. C.; Dijkstra E. J.; Achterop S.; Vondrumen R.; Vanderspoel D.; Sijbers A.; Keegstra H.; Renardus M. K. R. GROMACS - a Parallel Computer for Molecular-Dynamics Simulations; World Scientific Publishing: Singapore, 1993.

[ref47] Lindahl E.; Hess B.; van der Spoel D. GROMACS 3.0: A Package for Molecular Simulation and Trajectory Analysis. J. Mol. Model. 2001, 7, 306–317. 10.1007/s008940100045.

[ref48] Van Der Spoel D.; Lindahl E.; Hess B.; Groenhof G.; Mark A. E.; Berendsen H. J. C. GROMACS: Fast, Flexible, and Free. J. Comput. Chem. 2005, 26, 1701–1718. 10.1002/jcc.20291. 16211538

[ref49] Hess B.; Kutzner C.; van der Spoel D.; Lindahl E. GROMACS 4: Algorithms for Highly Efficient, Load-Balanced, and Scalable Molecular Simulation. J. Chem. Theory Comput. 2008, 4, 435–447. 10.1021/ct700301q. 26620784

[ref50] Abraham M. J.; Murtola T.; Schulz R.; Páll S.; Smith J. C.; Hess B.; Lindahl E. GROMACS: High Performance Molecular Simulations through Multi-Level Parallelism from Laptops to Supercomputers. SoftwareX 2015, 1–2, 19–25. 10.1016/j.softx.2015.06.001.

[ref51] Schmid N.; Eichenberger A. P.; Choutko A.; Riniker S.; Winger M.; Mark A. E.; van Gunsteren W. F. Definition and Testing of the GROMOS Force-Field Versions 54A7 and 54B7. Eur. Biophys. J. 2011, 40, 843–856. 10.1007/s00249-011-0700-9. 21533652

[ref52] Malde A. K.; Zuo L.; Breeze M.; Stroet M.; Poger D.; Nair P. C.; Oostenbrink C.; Mark A. E. An Automated Force Field Topology Builder (ATB) and Repository: Version 1.0. J. Chem. Theory Comput. 2011, 7, 4026–4037. 10.1021/ct200196m. 26598349

[ref53] Canzar S.; El-Kebir M.; Pool R.; Elbassioni K.; Malde A. K.; Mark A. E.; Geerke D. P.; Stougie L.; Klau G. W. Charge Group Partitioning in Biomolecular Simulation. J. Comput. Biol. 2013, 20, 188–198. 10.1089/cmb.2012.0239. 23461571 PMC3590896

[ref54] Koziara K. B.; Stroet M.; Malde A. K.; Mark A. E. Testing and Validation of the Automated Topology Builder (ATB) Version 2.0: Prediction of Hydration Free Enthalpies. J. Comput.-Aided Mol. Des. 2014, 28, 221–233. 10.1007/s10822-014-9713-7. 24477799

[ref55] Martínez L.; Andrade R.; Birgin E. G.; Martínez J. M. PACKMOL: A Package for Building Initial Configurations for Molecular Dynamics Simulations. J. Comput. Chem. 2009, 30, 2157–2164. 10.1002/jcc.21224. 19229944

[ref56] Brehm M.; Thomas M.; Gehrke S.; Kirchner B. TRAVIS—A Free Analyzer for Trajectories from Molecular Simulation. J. Chem. Phys. 2020, 152, 164105 10.1063/5.0005078. 32357781

[ref57] Brehm M.; Kirchner B. TRAVIS - A Free Analyzer and Visualizer for Monte Carlo and Molecular Dynamics Trajectories. J. Chem. Inf. Model. 2011, 51, 2007–2023. 10.1021/ci200217w. 21761915

[ref58] Hollóczki O.; Macchiagodena M.; Weber H.; Thomas M.; Brehm M.; Stark A.; Russina O.; Triolo A.; Kirchner B. Triphilic Ionic-Liquid Mixtures: Fluorinated and Non-Fluorinated Aprotic Ionic-Liquid Mixtures. ChemPhysChem 2015, 16, 3325–3333. 10.1002/cphc.201500473. 26305804 PMC4641458

[ref59] Grimme S. Density functional theory with London dispersion corrections. WIREs Comput. Mol. Sci. 2011, 1, 211–228. 10.1002/wcms.30.

[ref60] Neese F. The ORCA Program System. WIREs Comput. Mol. Sci. 2012, 2, 73–78. 10.1002/wcms.81.

[ref61] Neese F. Software Update: The ORCA Program System, Version 4.0. WIREs Comput. Mol. Sci. 2018, 8, e1327 10.1002/wcms.1327.

[ref62] Neese F. Software Update: The ORCA Program System—Version 5.0. WIREs Comput. Mol. Sci. 2022, 12, e1606 10.1002/wcms.1606.

[ref63] Bannwarth C.; Caldeweyher E.; Ehlert S.; Hansen A.; Pracht P.; Seibert J.; Spicher S.; Grimme S. Extended Tight-Binding Quantum Chemistry Methods. WIREs Comput. Mol. Sci. 2021, 11, e1493 10.1002/wcms.1493.

[ref64] Betlejewska-Kielak K.; Bednarek E.; Budzianowski A.; Michalska K.; Maurin J. K. Comprehensive Characterisation of the Flurbiprofen/β-Cyclodextrin Inclusion Complex Using X-Ray Techniques and NMR Spectroscopy. J. Mol. Struct. 2023, 1285, 135450 10.1016/j.molstruc.2023.135450. PMC8271474 34279429

[ref65] Tian B.; Feng Y.; Li X.; Yang J.; Ding Z.; Huang X.; Yin Q.; Xie C.; Hao H. Solution Thermodynamic Properties of Flurbiprofen in Twelve Solvents from 283.15 to 323.15 K. J. Mol. Liq. 2019, 296, 111744 10.1016/j.molliq.2019.111744.

[ref66] Grzesiak A. L.; Matzger A. J. New Form Discovery for the Analgesics Flurbiprofen and Sulindac Facilitated by Polymer-induced Heteronucleation. J. Pharm. Sci. 2007, 96, 2978–2986. 10.1002/jps.20954. 17567888 PMC2581769

[ref67] Tumanova N.; Tumanov N.; Robeyns K.; Fischer F.; Fusaro L.; Morelle F.; Ban V.; Hautier G.; Filinchuk Y.; Wouters J.; et al. Opening Pandora’s Box: Chirality, Polymorphism, and Stoichiometric Diversity in Flurbiprofen/Proline Cocrystals. Cryst. Growth Des. 2018, 18, 954–961. 10.1021/acs.cgd.7b01436.

[ref68] Liu D.; Liu Z.-R.; Wang Z.-H.; Ma C.; Herbert S.; Schirok H.; Mei T.-S. Paired Electrolysis-Enabled Nickel-Catalyzed Enantioselective Reductive Cross-Coupling between α-Chloroesters and Aryl Bromides. Nat. Commun. 2022, 13, 7318 10.1038/s41467-022-35073-z. 36443306 PMC9705544

[ref69] Sagdinc S.; Pir H. Spectroscopic and DFT Studies of Flurbiprofen as Dimer and Its Cu(II) and Hg(II) Complexes. Spectrochim. Acta, Part A 2009, 73, 181–194. 10.1016/j.saa.2009.02.022. 19285917

[ref70] Essa E. A.; Elmarakby A. O.; Donia A. M. A.; El Maghraby G. M. Controlled Precipitation for Enhanced Dissolution Rate of Flurbiprofen: Development of Rapidly Disintegrating Tablets. Drug Dev. Ind. Pharm. 2017, 43, 1430–1439. 10.1080/03639045.2017.1318905. 28402193

[ref71] Havriliak S.; Negami S. A Complex Plane Analysis of α-Dispersions in Some Polymer Systems. J. Polym. Sci., Part C: Polym. Symp. 1966, 14, 99–117. 10.1002/polc.5070140111.

[ref72] Vogel H. Das Temperaturabhangigkeitsgesetz Der Viskositat von Flussigkeiten. Phys. Z. 1921, 22, 645–646.

[ref73] Fulcher G. S. Analysis of Recent Measurements of the Viscosity of Glasses. J. Am. Ceram. Soc. 1925, 8, 339–355. 10.1111/j.1151-2916.1925.tb16731.x.

[ref74] Tammann G. A.; Hesse W. Die Abhängigkeit Der Viscosität von Der Temperatur Bie Unterkühlten Flüssigkeiten. Z. Anorg. Allg. Chem. 1926, 156, 245–257. 10.1002/zaac.19261560121.

[ref75] Ngai K. L.; Wang L.-M. Relations between the Structural α-Relaxation and the Johari–Goldstein β-Relaxation in Two Monohydroxyl Alcohols: 1-Propanol and 5-Methyl-2-Hexanol. J. Phys. Chem. B 2019, 123, 714–719. 10.1021/acs.jpcb.8b11453. 30601008

[ref76] Wojnarowska Z.; Adrjanowicz K.; Wlodarczyk P.; Kaminska E.; Kaminski K.; Grzybowska K.; Wrzalik R.; Paluch M.; Ngai K. L. Broadband Dielectric Relaxation Study at Ambient and Elevated Pressure of Molecular Dynamics of Pharmaceutical: Indomethacin. J. Phys. Chem. B 2009, 113, 12536–12545. 10.1021/jp905162r. 19694472

[ref77] Ngai K. L.; Casalini R.; Capaccioli S.; Paluch M.; Roland C. M. Do Theories of the Glass Transition, in Which the Structural Relaxation Time Does Not Define the Dispersion of the Structural Relaxation, Need Revision?. J. Phys. Chem. B 2005, 109, 17356–17360. 10.1021/jp053439s. 16853218

[ref78] Adrjanowicz K.; Pionteck J.; Paluch M. Isochronal Superposition and Density Scaling of the Intermolecular Dynamics in Glass-Forming Liquids with Varying Hydrogen Bonding Propensity. RSC Adv. 2016, 6, 49370–49375. 10.1039/C6RA08406K.

[ref79] Jesionek P.; Heczko D.; Hachuła B.; Kamiński K.; Kamińska E. High-Pressure Studies in the Supercooled and Glassy State of the Strongly Associated Active Pharmaceutical Ingredient—Ticagrelor. Sci. Rep. 2023, 13, 8890 10.1038/s41598-023-35772-7. 37264074 PMC10235114

[ref80] Avramov I. Pressure Dependence of Viscosity of Glassforming Melts. J. Non-Cryst. Solids 2000, 262, 258–263. 10.1016/S0022-3093(99)00712-7.

[ref81] Avramov I. Pressure and Temperature Dependence of Viscosity of Glassforming and of Geoscientifically Relevant Systems. J. Volcanol. Geotherm. Res. 2007, 160, 165–174. 10.1016/j.jvolgeores.2006.09.006.

[ref82] Adrjanowicz K.; Kaminski K.; Wojnarowska Z.; Dulski M.; Hawelek L.; Pawlus S.; Paluch M.; Sawicki W. Dielectric Relaxation and Crystallization Kinetics of Ibuprofen at Ambient and Elevated Pressure. J. Phys. Chem. B 2010, 114, 6579–6593. 10.1021/jp910009b. 20415466

