# Supplementary material for: Variation of Activation Volume as an Indicator of the Difference in Clusterization Phenomenon Induced by H-Bonding and F−Π Stacking Interactions in Enantiomers and a Racemate of Flurbiprofen
Source: J Phys Chem B. 2024 Apr 12;128(16):4021–32. doi: 10.1021/acs.jpcb.4c00582 (PMC11056992; doi:10.1021/acs.jpcb.4c00582)
Supplement: Supplementary file 1 — jp4c00582_si_001.pdf [file jp4c00582_si_001.pdf]

Variation of Activation Volume as an Indicator of the Difference in Clusterization Phenomenon Induced by H-bonding and F – Π stacking Interactions in Enantiomers and a Racemate of Flurbiprofen

Paulina Jesionek[a,b,*], Barbara Hachuła[a], Karolina Jurkiewicz[c], Patryk Włodarczyk[d], Marek Hreczka[d,e] Kamil Kamiński[c], Ewa Kamińska[b,*]

[a] *Institute of Chemistry, Faculty of Science and Technology, University of Silesia in Katowice, Szkolna 9, 40-007 Katowice, Poland*

[b] *Department of Pharmacognosy and Phytochemistry, Faculty of Pharmaceutical Sciences in Sosnowiec, Medical University of Silesia in Katowice, Jagiellonska 4, 41-200 Sosnowiec, Poland*

[c] *Institute of Physics, Faculty of Science and Technology, University of Silesia in Katowice, 75 Pulku Piechoty 1, 41-500 Chorzow, Poland*

[d] *Łukasiewicz Research Network - Institute of Non-Ferrous Metals, Sowinskiego 5 St., 44-100, Gliwice, Poland*

[e] *Department of Mechatronics, Silesian University of Technology, Akademicka 10A St., 44-100, Gliwice, Poland*

SUPPORTING INFORMATION

Results:

FTIR Spectroscopy data

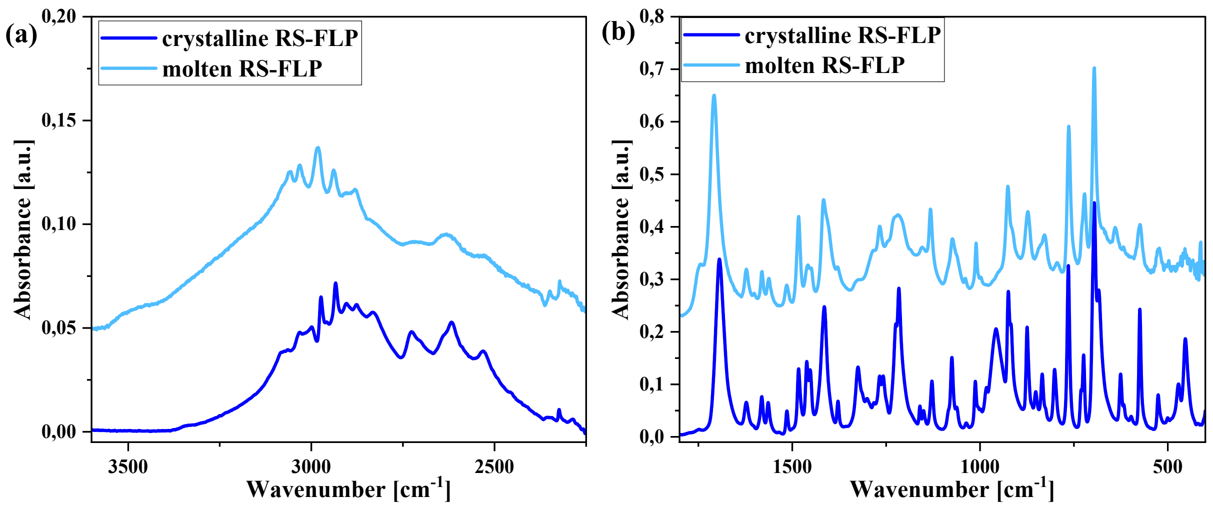

Figure S1. Comparison of FTIR spectra of crystalline and melted racemic FLP in the wavenumber regions of (a) 3600-2250 cm^{-1} and (b) 1800-400 cm^{-1}.

BDS data

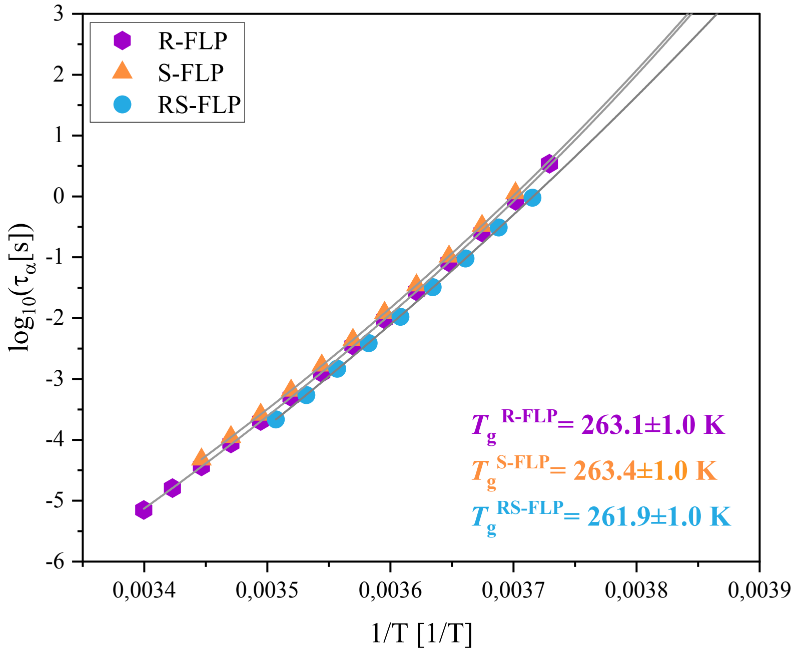

Figure S2. Temperature dependence of structural relaxation times determined from ambient pressure BDS measurements for *R*-, *S*-, and *RS*-FLP. The solid lines represent fits using the Vogel–Fulcher–Tammann (VFT) equation: $\tau_\alpha = \tau_{VFT}\exp\left(\frac{D_T T_0}{T-T_0}\right)$, where τ_{VFT} is the time scale of vibrational movements, D_T is the strength parameter, and T_0 represents the temperature at which relaxation times tend to infinity). The values of T_g correspond to the structural relaxation time of 100 s.

Table S1. Parameters of the modified Avramov equation (eq. 4) obtained from the analysis of $\tau_\alpha\,(T, p)$ dependencies for *R*-FLP, *S*-FLP, and *RS*-FLP.

	***R*-FLP**	***S*-FLP**	***RS*-FLP**
$\log_{10}(\tau_\infty[s])$	-14.02±1.16	-13.56 ± 1.27	-15.84 ± 2.03
T_r [K]	263.20±0.23	263.50 ± 0.23	261.49 ± 0.47
α_0	5.276±0.535	5.379 ± 0.602	4.372 ± 0.562
C/C_{p0}	0.082±0.007	0.001 ± 0.0001	0.062 ± 0.002
Π [MPa]	212.47±39.60	410.74 ± 86.22	354.97 ± 88.53
β	0.991±0.154	1.969 ± 0.292	1.338 ± 0.329
Adj. R-Square	0.998	0.999	0.999

Results of MD simulations and DFT computations

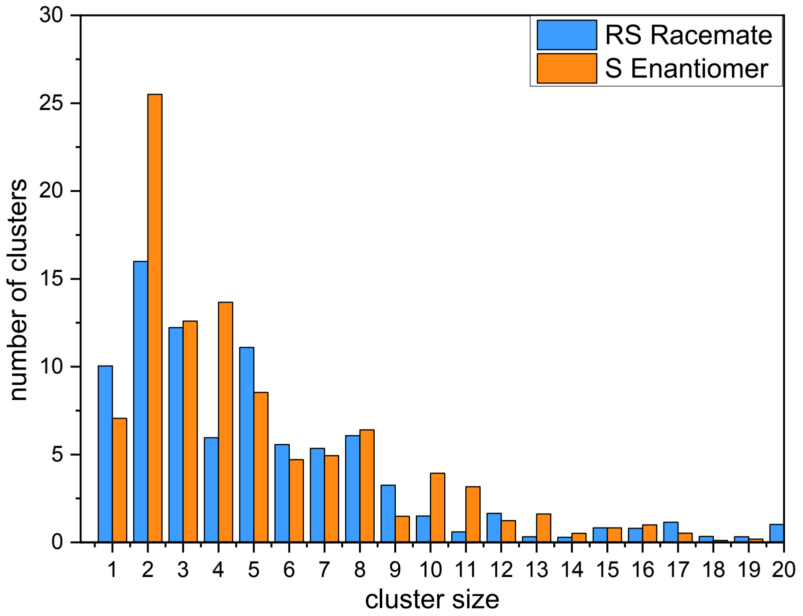

Figure S3. Histogram of HB clusters distribution in *RS*- and *S*-FLP systems.

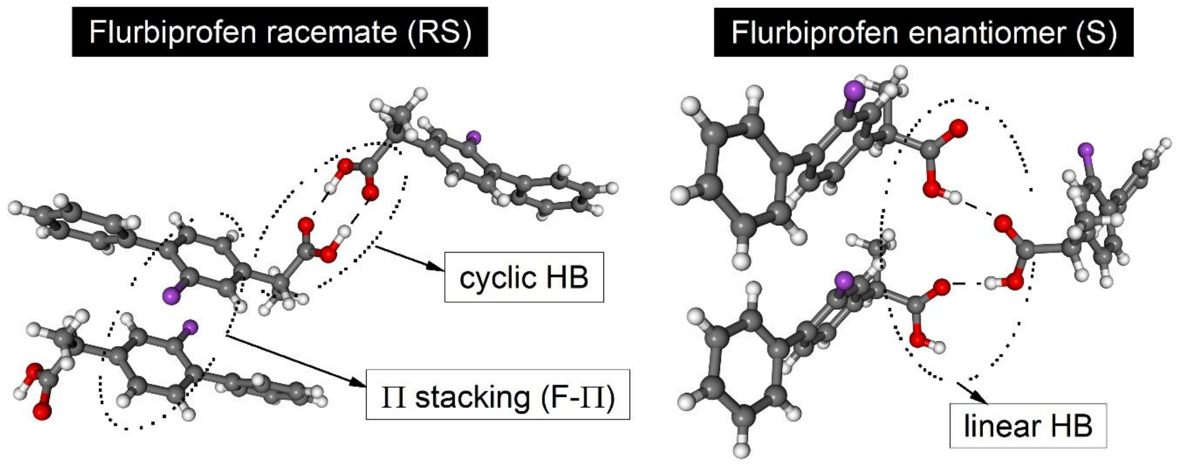

Figure S4. Comparison of flurbiprofen mer repeating units in the crystal structures. In the racemate, the (cHB ··· Π ··· cHB) pattern, while in the enantiomer, the (HB···HB···HB) pattern can be found in their crystalline states.
